# Proteasome activators, PA28γ and PA200, play indispensable roles in male fertility

**DOI:** 10.1038/srep23171

**Published:** 2016-03-22

**Authors:** Lin Huang, Kousuke Haratake, Hatsumi Miyahara, Tomoki Chiba

**Affiliations:** 1 Graduate School of Life and Environmental Sciences, University of Tsukuba, Tsukuba, Ibaraki 305-8577, Japan; 2 Department of Pathophysiology, Dalian Medical University, Dalian, Liaoning 116044, P.R. China

## Abstract

Protein degradation mediated by the proteasome is important for the protein homeostasis. Various proteasome activators, such as PA28 and PA200, regulate the proteasome function. Here we show double knockout (dKO) mice of *Psme3* and *Psme4* (genes for PA28γ and PA200), but not each single knockout mice, are completely infertile in male. The dKO sperms exhibited remarkable defects in motility, although most of them showed normal appearance in morphology. The proteasome activity of the mutant sperms decreased notably, and the sperms were strongly positive with ubiquitin staining. Quantitative analyses of proteins expressed in dKO sperms revealed up-regulation of several proteins involved in oxidative stress response. Furthermore, increased 8-OHdG staining was observed in dKO sperms head, suggesting defective response to oxidative damage. This report verified PA28γ and PA200 play indispensable roles in male fertility, and provides a novel insight into the role of proteasome activators in antioxidant response.

Protein turnover is an essential event in eukaryotic cells, which is regulated primarily by the proteasome. The 26S proteasome consists of two subcomplexes, the 20S catalytic particle (CP) and the regulatory particles (RPs) that bind at either or both ends of the 20S CP. 20S CP is a barrel-shaped complex made of two outer α-rings and two inner β-rings. The α- and β-rings are each made up of seven structurally similar α- and β-subunits, respectively. The proteolytic activity is exerted by the three β-subunits, β1, β2 and β5. Analysis of 20S CP structure indicates that the center of the α-ring is almost closed, thus preventing penetration of substrates into the interior of the β-ring on which the proteolytic active sites are located^1^.

20S CP is activated by association with various RPs, that open the α-ring, such as PA700, PA28 and PA200. PA700, the 19S activator, fulfills the roles of the proteasome in ubiquitin-dependent protein degradation. It contains subunits that can capture ubiquitinated proteins and ATPase subunits that unfold the captured substrate. On the other hand, PA28 and PA200 do not recognize ubiquitinated protein nor possess ATPase activity. PA28 is encoded by three homologous genes, namely *Psme1* (PA28α), *Psme2* (PA28β) and *Psme3* (PA28γ). PA28α and PA28β preferentially form a heteroheptamer with nearly equal stoichiometric amounts in the cytosol, while PA28γ forms a homoheptamer and localizes in nucleus. Association of PA28 with the 20S CP greatly stimulates multiple peptidase activities of the 20S CP *in vitro*, without requiring energy. To date, only a few protein substrates of the PA28γ-proteasome pathway have been identified, which include steroid receptor coactivator 3 (SRC3)^2^, hepatitis C virus core protein^3^, the tumor suppressor p53^4^, the cell cycle inhibitors p21 (also known as CIP1 or WAF1)^5^,^6^, p16 (also known as INK4A)^6^, and p19 (also known as ARF)^6^. These substrates are degraded through both ubiquitin- dependent and independent ways.

Similar as PA28, PA200, which is encoded by *Psme4*, stimulates the hydrolysis of small peptides or the unstructured tau protein *in vitro* without utilizing ATP^7^,^8^. PA200 is present in all mammalian tissues, and is highly expressed in testis^9^ and sperm^10^. The *Psme4*-deficient mice show defects in normal spermatogenesis^11^, DNA repair and acetylation-dependent degradation of histones^12^.

Spermatogenesis is a complicated developmental program that includes remodeling of the haploid round spermatids to elongated spermatids. Spermatids released from the testis undergo further maturation during transport through the epididymis^13^. The formation of mature sperm is precisely regulated, which depends significantly on the appropriate timing of expression of specific proteins that play critical roles in mediating these events^14^,^15^. Besides transcriptional and translational regulation, the protein degradation controlled by the proteasome is also critical for the protein expression. Despite the spermatogenesis defects, *Psme4* knockout (KO) male mice are still fertile. Since PA200 and other RPs may have redundant roles to activate the proteasome, here we constructed *Psme3/Psme4* double KO (dKO) mice, and found that the dKO male mice are completely infertile. The *Psme3/Psme4* dKO male sperms had significantly reduced motility and proteasome activity. Quantitative mass spectrometry analysis of the mutant sperms indicates increased expression of proteins involved in redox balance. These results provide a novel insight into the role of the proteasome in antioxidant response and male fertility.

## Results

### *Psme3/Psme4* dKO male mice are completely infertile

KO mice for *Psme3*^16^ and *Psme4*^12^ in the same C57BL/6 J genetic background were independently maintained by successful breeding. We then generated *Psme3* heterozygous mice in *Psme4*-deficient background (*Psme3*^+/−^*Psme4*^*−/−*^) and *Psme4* heterozygous mice in *Psme3*-deficient background (*Psme3*^*−/−*^*Psme4*^+/−^). Both mutant strains did not manifest significant abnormality in fertility and were each intercrossed to obtain dKO mice for *Psme3* and *Psme4*. *Psme3*^*−/−*^*Psme4*^*−/−*^ dKO mice were born at predicted Mendelian ratios (Supplementary Table S1), which suggest that the simultaneous deficiency of *Psme3* and *Psme4* does not result in mortality or severe developmental defects during embryogenesis.

When 9 dKO male mice were mated to total 28 females, none of them could impregnate the females, while all *Psme3*^+/−^*Psme4*^*−/−*^ and *Psme3*^*−/−*^*Psme4*^+/−^ littermate controls could (Tables 1, 2, [Table t1], [Table t1], [Table t2]). These results indicate that the dKO male is completely infertile, and suggest PA200 and PA28γ additively or synergistically play indispensable roles in male fertility.

### The *Psme3/Psme4* dKO sperms are immotile

To analyze the infertility of *Psme3/Psme4* dKO male mice, we first investigated the testis histology. *Psme3*^+/−^*Psme4*^*−/−*^ and *Psme3*^*−/−*^*Psme4*^+/−^ littermate controls and wild type (WT) males at the same age, were used as controls. The size of the mutant testis was comparable to that of the control littermates, and histological analyses revealed significant number of apparently normal sperm in the seminiferous tubules (Supplementary Fig. S1). We next isolated sperms from cauda epididymis (Supplementary Fig. S2a–c). It is previously reported that *Psme4* KO mice have increased giant round cells^11^. Indeed, *Psme3*^+/−^*Psme4*^*−/−*^ mice had increased incidence of giant round cells compared to WT mice, which was 0.3 ± 0.2% and 0.2 ± 0%, respectively. In *Psme3/Psme4* dKO mice, the incidence rose to 2.7 ± 0.1% (Supplementary Fig. S2a,b). Head only cells also increased to 20.7 ± 1.9% in *Psme3/Psme4* dKO mice. These results suggest abnormal spermatogenesis in dKO male mice. However, a significant number of normal sperms were still observed in dKO mice (dKO 76.6 ± 7.1% vs WT 93.3 ± 5.4%) (Supplementary Fig. S2a,b). Similar results were obtained in the experiments using littermates from *Psme3*^*−/−*^*Psme4*^+/−^ breeding pairs (Supplementary Fig. S2c). Therefore, it was unlikely that increased number of abnormal sperms could explain the complete infertility of dKO male mice.

**Figure 1:**
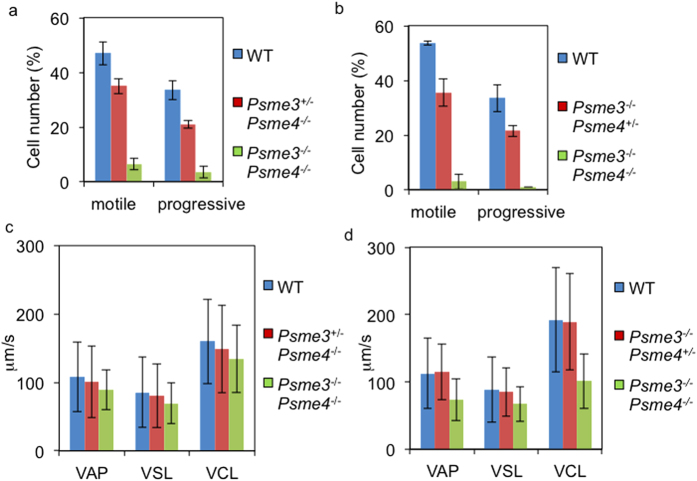
Sperm motility of *Psme3/Psme4* dKO mice. Sperms isolated from the cauda epididymis of wild type (WT) (**a**–**d**), *Psme3*^+/−^*Psme4*^*−/−*^ (**a**,**c**), *Psme3*^*−/−*^*Psme4*^+/−^ (**b**,**d**) and *Psme3*^*−/−*^*Psme4*^*−/−*^ (**a**–**d**) mice were incubated in TYH drops for 1.5 h, and subjected to computer assisted sperm analysis (CASA). VAP, average path velocity; VSL, straight line velocity; VCL, curvilinear velocity. Bars, SD.

We next incubated the isolated sperms in TYH culture drops. Both WT and the littermate controls sperms started to swim and diffused throughout the culture drops within 15 min. In contrast, most of *Psme3/Psme4* dKO sperms could not diffuse and remained as clumps even after 1.5 h of incubation (Supplementary Fig. S3). We then applied the sperms to computer assisted sperm analysis (CASA). For the WT sperms, the ratios of motile and progressive sperms were 53.5 ± 0.7% and 33.5 ± 5.0%, respectively. The ratio of motile sperms in *Psme3*^*−/−*^*Psme4*^+/−^ and *Psme3*^+/−^*Psme4*^*−/−*^ littermate controls were 35.5 ± 4.9% and 35.0 ± 2.8%, respectively, and those of progressive sperms were 21.5 ± 2.1% and 21.0 ± 1.4%, respectively. On the other hand, the ratios of motile and progressive sperms of the *Psme3/Psme4* dKO mice were significantly reduced to 3.0 ± 2.8% and 1.0 ± 0%, which indicates that only a few sperm could acquire motility in the dKO mice (Fig. 1a,b). Those few motile progressive sperms in the *Psme3/Psme4* dKO sperms had normal average path velocity (VAP), straight-line velocity (VSL) and curvilinear velocity (VCL) compared with controls (Fig.1c,d). These results suggest that the *Psme3/Psme4* dKO sperms have functionally intact fragellum and its molecular motors are not impaired.

### *Psme3/Psme4* dKO sperms can fertilize eggs *in vitro*

**Figure 2:**
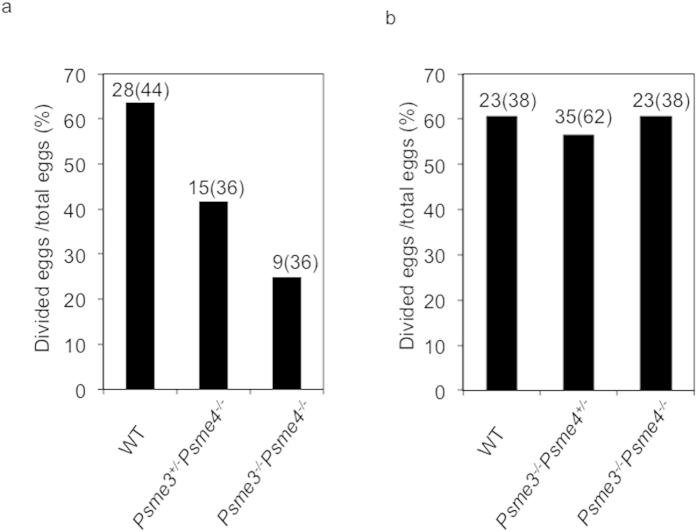
*In vitro* fertilization of *Psme3/Psme4* dKO sperms. Sperms isolated from the cauda epididymis of wild type (WT) (**a**,**b**), *Psme3*^+/−^*Psme4*^*−/−*^ (**a**), *Psme3*^*−/−*^*Psme4*^+/−^ (**b**) and *Psme3*^*−/−*^*Psme4*^*−/−*^ mice (**a**,**b**) were incubated in TYH media for 1.5 h. Sperms were co-cultured with eggs and fertilized eggs were counted 48 h later. The numbers of the fertilized eggs and total eggs in parentheses are shown on each column. The experiments were repeated three times, and the values from a representative experiment are shown.

Although *Psme3/Psme4* dKO sperms were mostly immotile, a few sperm could acquire the progressive motility. Thus we further asked whether *Psme3/Psme4* dKO sperms can *in vitro* fertilize (IVF) the eggs. The few progressive sperms collected from the border of TYH culture drops were put near the eggs. As the results, successful fertilization was observed in *Psme3/Psme4* dKO sperms although the fertilization was not efficient compared with controls (Fig. 2). These data indicate that the sperms of *Psme3/Psme4* dKO male can complete acrosome reaction. Taken together, the main reason of *Psme3/Psme4* dKO male infertility is due to its inefficient ability to acquire the motility.

### Proteasome activity is decreased in *Psme3/Psme4* dKO sperms

**Figure 3:**
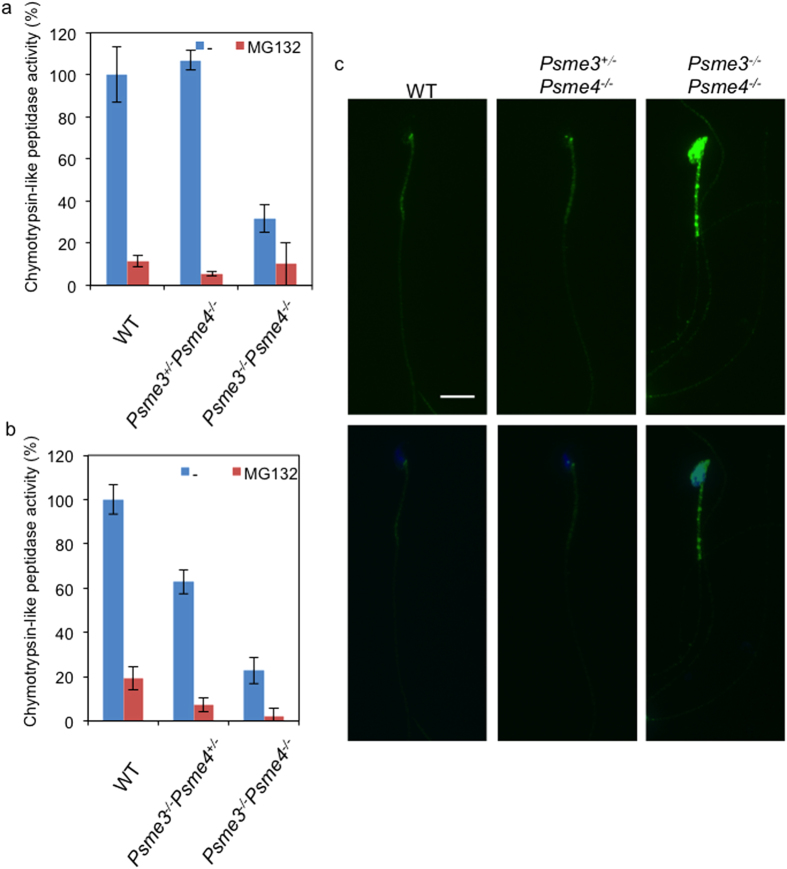
Proteasome activity of *Psme3/Psme4* dKO sperms. (**a**,**b**) 2 × 10^5^ sperms of wild type (WT) (**a**,**b**), *Psme3*^+/−^*Psme4*^*−/−*^ (**a**), *Psme3*^*−/−*^ *Psme4*^+/−^ (**b**) and *Psme3*^*−/−*^*Psme4*^*−/−*^ (**a**,**b**) mice were inoculated into 96-well plate, treated with or without 5 μM of MG132 and the proteasome peptidase activities were measured. The activities relative to WT are shown. Bars, SD. (**c**) Immunostaining of sperms by anti-multiubiquitin antibody. Green, multiubiquitin; Blue, Hoechst 33342. Scale bar, 10 μm.

Next, we investigated the proteasome activity of the sperm by measuring the chymotrypsin-like activity. The chymotrypsin-like activity of the proteasome in the *Psme3/Psme4* dKO sperms was remarkably down-regulated to 20–30% of that of WT sperms, whereas the activities of heterozygote *Psme3*^+/−^*Psme4*^*−/−*^ and *Psme3*^*−/−*^*Psme4*^+/−^ littermate control were comparable or reduced to 60% of that of WT sperms (Fig. 3a,b).

Given that the proteasome is important for the degradation of ubiquitinated proteins, we next investigated the level of ubiquitinated proteins in the sperms by immunohistochemistry. The level of ubiquitin-positive staining was higher in *Psme3/Psme4* dKO sperms compared to WT and *Psme3*^+/−^*Psme4*^*−/−*^ littermate control sperms (Fig. 3c), which was consistent with the decrease of proteasome activity (Fig. 3a). Therefore, not only the decrease of proteasome activity, but also the elevated levels of ubiquitinated proteins, especially the proteins that should be degraded by either PA28γ- or PA200-dependent pathways may play a crucial role in the regulation of male fertility.

### Gpx5 level increases in *Psme3/Psme4* dKO mice

**Figure 4:**
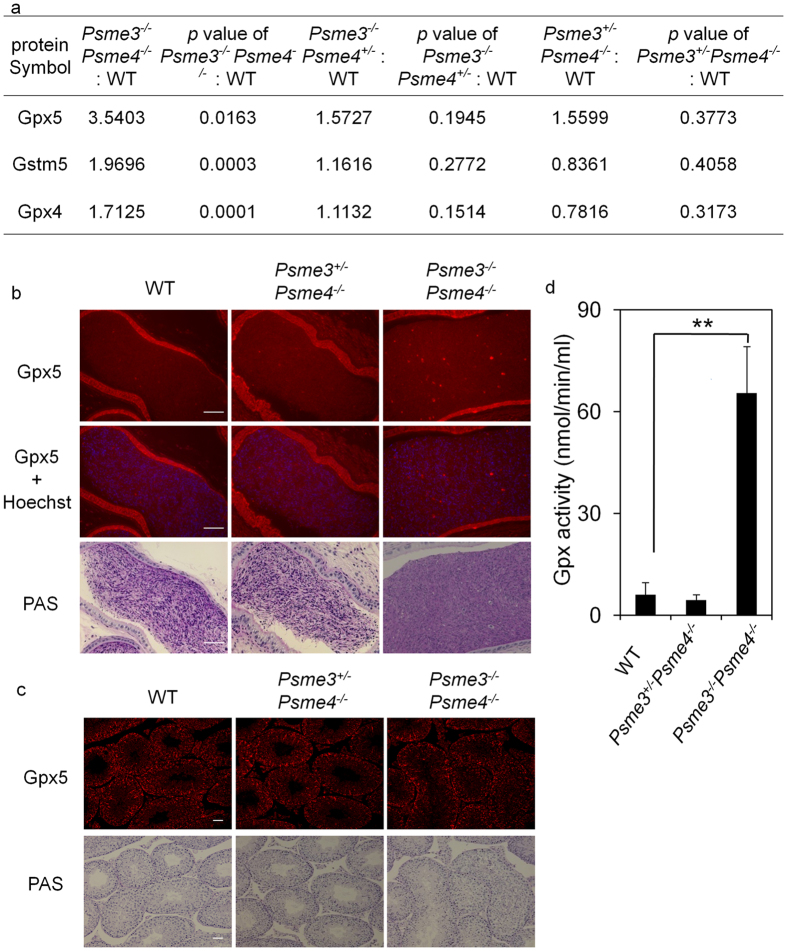
Gpx5 expression in *Psme3/Psme4* dKO sperms. (**a**) Proteins significantly enriched in *Psme3*^*−/−*^*Psme4*^*−/−*^ sperms were identified from two independent LC/MS/MS analyses. The relative amounts of proteins and *p* values in *Psme3*^*−/−*^*Psme4*^*−/−*^, *Psme3*^*−/−*^*Psme4*^+/−^ or *Psme3*^+/−^*Psme4*^*−/−*^ to WT sperms from the representative experiment are shown. (**b**,**c**) Gpx5 immunostaining (upper panels) and PAS-Hematoxylin staining (lower panels) in serial sections of cauda epididymis (**b**) and testes (**c**). Red, Gpx5; Blue, Hoechst 33342. Scale bars, 50 μm. (**d**) Total Gpx activity in cauda epididymis lysate. The experiments were conducted in duplicate. Bars, SD. **p < 0.01 by Student’s *t* test.

As reduced proteasome activity and enhanced ubiquitin positive signal were observed in *Psme3/Psme4* dKO sperms, we sought to identify the proteins specifically accumulated in the dKO sperms. To this end, we extracted proteins from sperms and analyzed the proteins up-regulated in *Psme3/Psme4* dKO mice by quantitative mass spectrometry. The proteins significantly increased only in *Psme3/Psme4* dKO sperms in two independent experiments were identified according to the following criteria; the difference between *Psme3*^*−/−*^*Psme4*^*−/−*^ and WT levels is significant *p* < 0.05, while those with littermate controls (*Psme3*^*−/−*^*Psme4*^+/−^ and *Psme3*^*+/−*^*Psme4*^*−/−*^) are insignificant *p* > 0.05 (Supplementary Tables 3 and 4). Interestingly, the identified proteins that increased only in *Psme3/Psme4* dKO sperms, but not littermate controls, were all related to glutathione metabolism, which protect cells from oxidative attacks (Fig. 4a). Among them, Gpx5, which is highly expressed in male reproduction tract, showed the highest increase in dKO sperms. Therefore we focused on Gpx5 for further analyses.

Gpx5 belongs to glutathione (GSH) peroxidase family, which catalyzes the reduction of a variety of hydroperoxides including lipid peroxides, using GSH as a specific electron donor substrate. Gpx5 expression is regulated by androgen, and is specifically expressed in epididymis and epididymosome^17^,^18^,^19^. Gpx5 in the epididymosome^20^, has been proposed to play a role in protecting the membranes of sperms from the damaging effects of lipid peroxidation and/or preventing premature acrosome reaction^21^. To analyze the expression of Gpx5, we immunostained the testes and cauda epididymis with anti-Gpx5 antibody. Although the signal of Gpx5 staining was virtually normal in the mutant testes, it was higher in cauda epididymis of mutant mice. Furthermore, strong Gpx5 signal was observed in an abnormal structure, which was scarcely observed in the control samples (Fig. 4b and Supplementary Fig. S5). The total Gpx activity was further analyzed using cauda epididymis lysate (Fig. 4d). In agreement with the quantitative mass spectrometry and immunostaining data, the dKO mice showed a significant increase in total Gpx activity compared with WT and *Psme3*^+/−^*Psme4*^*−/−*^ control mice (Fig. 4d). These abnormalities in the cauda epididymis suggest the link between antioxidant response and *Psme3/Psme4* deficiency.

### *Psme3/Psme4* dKO sperms suffer from oxidative damages

**Figure 5:**
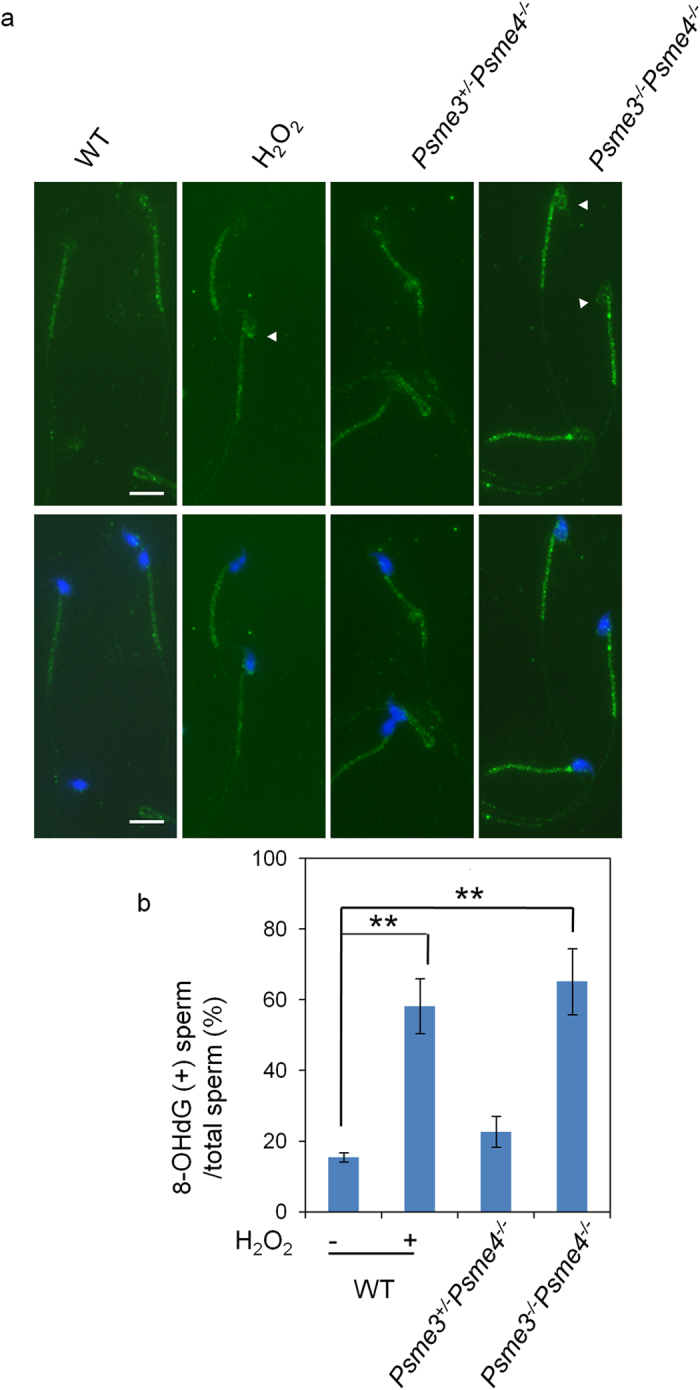
8-OHdG staining of the sperms. (**a**) Representative 8-OHdG staining of sperms isolated from WT mice (WT) treated without or with 100 μM H_2_O_2_ (H_2_O_2_), *Psme3*^+/−^*Psme4*^*−/−*^ and *Psme3*^*−/−*^*Psme4*^*−/−*^ mice. Green, 8-OHdG; Blue, Hoechst 33342; arrow heads, 8-OHdG-positive sperm heads. Scale bars, 10 μm. (**b**) The percentage of 8-OHdG-positive sperms. 200 sperms for each sample were observed and the percentages of sperm with 8-OHdG-positive head were calculated. The results of three independent experiments are shown. The sperms of WT mice treated with H_2_O_2_ were used as a positive control. Bars, SD. **p < 0.01 by Student’s *t* test.

The proteins increased in *Psme3/Psme4* dKO sperms were all related to oxidative stress response. Since oxidative stress causes DNA damages, we analyzed the level of 8-hydroxy-deoxyguanosine (8-OHdG) on sperm DNA. In the WT sperms, 8-OHdG staining was mainly observed in the tail but not in the head. The positive staining in the head appeared when the sperms were treated with hydrogen peroxide (Fig. 5a), suggesting that the DNA damages induced by oxidative attack can be detected in part by 8-OHdG staining. Then, the level of 8-OHdG was analyzed in *Psme3/Psme4* dKO sperms. The dKO sperms showed a higher signal in the sperm head, similar as that of the positive control treated with hydrogen peroxide (Fig. 5a). The heterozygous *Psme3*^+/−^*Psme4*^*−/−*^ control mice did not show such staining. The incidence of 8-OHdG positive sperm in *Psme3/Psme4* dKO sperms (65.2 ± 9.4%) was significantly higher than those of WT (15.4 ± 1.4%) and *Psme3*^+/−^*Psme4*^*−/−*^ littermate control mice (22.7 ± 4.4%), and its level was comparable to H_2_O_2_-treated WT sperms (58.2 ± 7.8%; Fig. 5b).

Since the dKO sperms appear to suffer from oxidative damage, we treated the sperms with the antioxidant, GSH or EDTA. In WT sperms, H_2_O_2_ treatment reduced the motility of the sperm and addition of GSH significantly restored the motility (Supplementary Fig. S6), suggesting that excess oxidative stress impairs the sperm motility. On the other hand, the motility of dKO sperm was not reversed by either GSH or EDTA treatment, suggesting that the defects of dKO sperm are either irreversible and/or associated with other abnormalities such as accumulation of ubiquitinated proteins.

## Discussion

Many diseases are results of homeostasis disturbance. In eukaryotic cells, protein degradation is mainly controlled by the proteasome, which rapidly degrades misfolded, oxidized and damaged proteins. The activity of the proteasome is controlled by various RPs, which can be subdivided into ATP-dependent and independent particles. The former particle can recognize ubiquitinated proteins, while the latter RPs do not. The essential roles of the latter particles have not been well characterized because each single KO mice have no obvious severe phenotypes^11^,^12^,^16^,^22^,^23^. Although *Psme3* and *Psme4* KO mice are reported to have some abnormalities in spermatogenesis^11^,^12^,^24^, both KO mice are fertile. In this report we showed that the *Psme3/Psme4* dKO male mice are completely infertile (Tables 1, 2, [Table t1], [Table t1], [Table t2]). These results strongly suggest that PA28γ and PA200 can compensate to each other and play indispensable role(s) in male fertility. PA28γ has 2 other family members in mammal, PA28α and PA28β. Interestingly, triple KO mice of *Psme1, Psme2* and *Psme4* were fertile and could propagate normally, suggesting that PA28γ has important roles that cannot be compensated by PA28α or PA28β (unpublished data).

Why *Psme3/Psme4* dKO male mice are completely infertile? We did not find any severe morphological defects in testes histology (Supplementary Fig. S1, Fig. 4c). Furthermore, more than 70% of sperms in cauda epididymis were found to be normal in morphology (Supplementary Fig. S2). However, the sperms isolated from cauda epididymal tract could not acquire the motility efficiently (Fig. 1a,b). More than 90% of sperms remained immotile and only a few sperms acquired the progressive motility (Fig. 1). The few progressive *Psme3/Psme4* dKO sperms were still able to fertilize the egg at least *in vitro* (Fig. 2). Therefore the infertility of the mutant mice is due to the defect in acquiring the motility, and the proteasome activators are not critically important for the acrosome reaction.

Sperms entering the caput epididymis lack the ability to swim and fertilize eggs at this stage. During their transit into the epididymis, sperms undergo maturation processes called spermiogenesis to acquire those functions. Therefore the defects in *Psme3/Psme4* dKO sperms may be associated with abnormality in the epididymis and spermiogenesis. It is well known that numerous proteins are degraded by ubiquitin and proteasome system during spermiogenesis^25^,^26^. We found significant decrease of proteasome activity (Fig. 3), which was accompanied with strong ubiquitin-positive signals in the dKO sperm. Therefore we hypothesized that impaired acquisition of motility is in part due to the decrease of proteasome activity and subsequent accumulation of ubiquitin-positive proteins. In an attempt to identify such proteins, we performed a quantitative proteomic analyses of sperms, and found Gpx5 as a candidate protein. Gpx5 level was higher and showed abnormal appearance in the cauda epididymis of *Psme3/Psme4* dKO mice (Fig. 4, Supplementary Fig. S5). Gpx5 is an epididymosomal protein secreted into epididymal lumen by epididymal cells. Gpx5-containing epididymosomes are known to attach to sperm membrane when sperms pass the caput epididymis^20^. Gpx5 functions as an epididymal luminal H_2_O_2_ scavenger^27^ and *Gpx5*^*−/−*^ sperm DNA showed an increased sensitivity to oxidative attack^28^. Because the 8-OHdG signal was higher in *Psme3/Psme4* dKO sperms (Fig. 5), it is tempting to speculate that elevation of Gpx5 reflects some defect in antioxidant response. Although Gpx5 may have accumulated as specific target substrate, it could be also up-regulated as a compensatory adaptation response against excessive oxidative stress. It is known that PA28γ increases during H_2_O_2_-adaptation, and purified PA28γ significantly increases the ability of 20S proteasome to selectively degrade oxidized proteins *in vitro*^29^. Therefore *Psme3/Psme4* dKO mice might have failed to dispose oxidized proteins, and led to accumulation of such damaged and ubiquitinated proteins. The results of 8-OHdG staining also support our notion that *Psme3/Psme4* dKO sperm cannot efficiently dispose the oxidatively damaged proteins. As oxidative damage caused by H_2_O_2_ treatment can inhibit the sperm motility, the immobility of dKO sperm could be caused at least in part by defective disposal of oxidatively damaged protein. The GSH treatment, however, could not restore the defect of dKO sperm, suggesting that other abnormalities such as accumulation of ubiquitinated protein is also involved.

In this study, we demonstrated that *Psme3/Psme4* dKO male mice are completely infertile. This is the first report that suggests ATP- and ubiquitin- independent RPs have indispensable roles that PA700 cannot compensate for. The dKO sperms had increased levels of oxidative damage and ubiquitinated proteins. It is reported that PA28γ, PA200, and PA700 can all degrade oxidatively damaged proteins. Therefore, there may exist some specific condition(s) that PA700 cannot function during spermiogenesis. It is unlikely that deletion of *Psme3* and *Psme4* causes the impairment of PA700 function in general, because dKO mice were viable and there was no obvious defect in embryogenesis. On the other hand, PA28γ and PA200 can form hybrid proteasomes with PA700. Thus, the reduction of proteasome activity and accumulation of ubiquitinated protein may be caused by the dysfunction of the hybrid proteasome. In this regard, it is tempting to speculate that sperm specific proteasome has higher preference to PA28γ and PA200, compared to other RPs. Further analysis is required to reveal these possibilities. Although the molecular mechanism how PA28γ and PA200 regulate sperm motility remains to be elucidated, our results pose the possibility that PA28γ and PA200 are valuable targets to develop contraceptives without harmful side effects.

Considering that PA200 is important for the degradation of acetylated histone during spermatogenesis and DNA repair^12^, the dKO of *Psme3* and *Psme4* may lead to aberrant epigenetic regulation and increased mutagenesis of the sperm. Further analyses in these lines, would reveal the important physiological function of the ATP- and ubiquitin-independent RPs, which are not essential for cell viability, but are conserved across species.

## Methods

### Animal Experiments

All animal experiments were approved by the animal care and use committee of Graduate School of Life and Environmental Sciences, The University of Tsukuba. Methods were carried out in accordance with the approved guidelines of the University of Tsukuba.

### Antibodies

Anti-multi ubiquitin (MBL, Japan), anti-8-OHdG (Immundiagnostik AG, Bensheim, Germany), anti-Gpx5 (Proteintech, Chicago, IL), and anti-20S proteasome β5 (Santa cruz, Dallas, TX) antibodies were purchased.

### Production of *Psme3/Psme4* Double Knockout Mice

*Psme3* and *Psme4* knockout mice were described before^12^,^16^. *Psme3* and *Psme4* mutant mice were intercrossed to obtain *Psme3*^+/−^*Psme4*^*−/−*^ and *Psme3*^*−/−*^*Psme4*^+/−^ mice. *Psme3*^*−/−*^*Psme4*^*−/−*^ mice were obtained by intercrossing each *Psme3*^+/−^*Psme4*^*−/−*^ and *Psme3*^*−/−*^*Psme4*^+/−^ breeding pairs.

### Genotyping of Mice by PCR

Genomic DNAs extracted from tail tips were subjected to PCR reactions. The wild type and mutant alleles for each gene were detected in a multiplex PCR reaction using two sets of primers (Supplementary Fig. S4). The primers used in this study are listed in Supplementary Table S2.

### Fertility Test

Male littermates at 2–6 month-old were individually caged with 1-2 females. Caged females were changed every month or when pregnancy was confirmed. Separated females were kept for another 3 weeks to observe the delivery.

### Sperm Migration Assay

Sperms isolated from cauda epididymis were incubated in TYH medium for 1.5 h. Sperms on the border of the culture drops were collected and applied to computer assisted sperm analysis (CASA).

### *In Vitro* Fertilization

*In vitro* fertilization was performed as described^30^. In brief, eggs collected from super ovulated females were placed in TYH medium. Epididymal sperms collected from 4–6 month-old male mice were capacitated in TYH medium for 1.5 h at 37 °C, 5% CO_2_. Capacitated sperms were added into the TYH culture drops containing eggs at a final concentration of 4 × 10^5^/ml. Eight hours later, the eggs were replaced into M16 medium and incubated for another 24–48 h. The 2-cell- and 4-cell-stage eggs were counted as fertilized eggs.

### Sperm Proteasome Activity

The chymotrypsin-like activity of the proteasome in intact sperm was analyzed using the fluorescent substrate (Suc-LLVY-MCA). 2 × 10^5^ intact sperms were incubated in TYH medium without BSA, and 50 μmol/L fluorescent substrate for 30 min at 37 °C. The fluorescence was measured with excitation at 380 nm and emission at 460 nm using a spectrofluorometer (Perkin Elmer).

### Immunohistochemistry

Fresh sperms collected from the epididymis were washed with PBS, decondensed in decondensing buffer (2 mM DTT, 0.05% Triton in PBS), incubated with first antibody overnight at 4 °C, and Alexa-conjugated second antibody following PBS washing. Sperms were mounted on glass slides and observed under a fluorescence microscope (Biorevo, BZ-9000, Keyence).

Cauda epididymis and testes were fixed in Bouin’s fluid for PAS staining, and Farmer’s fluid for immunohistochemical staining.

### Mass Spectrometry

Sperm lysates were extracted in 50 μL of lysis buffer (8 M Urea, 0.2 M Ammonium Bicarbonate) with sonication. Each sperm lysate was trypsin-digested and labeled with iTRAQ 4-plex reagent (AB SCIEX) according to the manufacture’s instruction. The labeled peptides were applied to QTRAP^®^4500 LC/MS/MS System (AB SCIEX). These experiments were performed in duplicate. Identification and quantification of proteins from sperm lysates were performed using the ProteinPilot software, Version 4.0 (AB SCIEX).

### Gpx activity assay

The cauda epididymis lysates of each genotype of mice were extracted and their Gpx activities were measured using Glutathione Peroxidase Assay Kit (Abcam, Cambridge, UK) according to the manufacturer’s protocol. The experiments were conducted in duplicate.

## Additional Information

**How to cite this article**: Huang, L. *et al*. Proteasome activators, PA28γ and PA200, play indispensable roles in male fertility. *Sci. Rep*. **6**, 23171; doi: 10.1038/srep23171 (2016).

## Supplementary Material

Supplementary Information

## Figures and Tables

**Table 1 t1:** Male fertility and litter size obtained from *Psme3*^+/−^*Psme4*^*−/−*^ and *Psme3^*−/−*^Psme4^−/−^* male mice.

Male Genotype	Fertility	Litter Size
*Psme3*^+/−^*Psme4*^*−/−*^ (n = 3)△	3/3	4.1 ± 2.0*
*Psme3*^*−/−*^*Psme4*^*−/−*^ (n = 3)▲	0/3	0

^△^Total 7 females were crossed with 3 males.

^▲^Total 12 females were crossed with 3 males.

^*^Litter size is the mean ± SD of 7 litters.

**Table 2 t2:** Male fertility and litter size obtained from *Psme3*^*−/−*^*Psme4*^+/−^ and *Psme3^*−/−*^Psme4^−/−^* male mice.

Male Genotype	Fertility	Litter Size
*Psme3*^*−/−*^*Psme4*^+/−^ (n = 5)△	5/5	3.5 ± 2.3*
*Psme3*^*−/−*^*Psme4*^*−/−*^ (n = 6)▲	0/6	0

^△^Total 7 females were crossed with 5 males.

^▲^Total 16 females were crossed with 6 males.

^*^Litter size is the mean ± SD of 6 litters.

## References

[b1] Stadtmueller B. M. & Hill C. P. Proteasome activators. Mol Cell 41, 8–19 (2011). 21211719 10.1016/j.molcel.2010.12.020 PMC3040445

[b2] Li X. . The SRC-3/AIB1 coactivator is degraded in a ubiquitin- and ATP-independent manner by the REGgamma proteasome. Cell 124, 381–392 (2006). 16439211 10.1016/j.cell.2005.11.037

[b3] Moriishi K. . Proteasome activator PA28gamma-dependent nuclear retention and degradation of hepatitis C virus core protein. J Virol 77, 10237–10249 (2003). 12970408 10.1128/JVI.77.19.10237-10249.2003 PMC228494

[b4] Zhang Z. & Zhang R. Proteasome activator PA28 gamma regulates p53 by enhancing its MDM2-mediated degradation. EMBO J 27, 852–864 (2008). 18309296 10.1038/emboj.2008.25 PMC2265109

[b5] Li X. . Ubiquitin- and ATP-independent proteolytic turnover of p21 by the REGgamma-proteasome pathway. Mol Cell 26, 831–842 (2007). 17588518 10.1016/j.molcel.2007.05.028

[b6] Chen X., Barton L. F., Chi Y., Clurman B. E. & Roberts J. M. Ubiquitin-independent degradation of cell-cycle inhibitors by the REGgamma proteasome. Mol Cell 26, 843–852 (2007). 17588519 10.1016/j.molcel.2007.05.022 PMC2031223

[b7] Dange T. . Blm10 protein promotes proteasomal substrate turnover by an active gating mechanism. J Biol Chem 286, 42830–42839 (2011). 22025621 10.1074/jbc.M111.300178 PMC3234834

[b8] Schmidt M. . The HEAT repeat protein Blm10 regulates the yeast proteasome by capping the core particle. Nat Struct Mol Biol 12, 294–303 (2005). 15778719 10.1038/nsmb914

[b9] Ustrell V., Pratt G., Gorbea C. & Rechsteiner M. Purification and assay of proteasome activator PA200. Methods Enzymol 398, 321–329 (2005). 16275339 10.1016/S0076-6879(05)98026-9

[b10] Skerget S. . The Rhesus macaque (Macaca mulatta) sperm proteome. Mol Cell Proteomics 12, 3052–3067 (2013). 23816990 10.1074/mcp.M112.026476 PMC3820923

[b11] Khor B. . Proteasome activator PA200 is required for normal spermatogenesis. Mol Cell Biol 26, 2999–3007 (2006). 16581775 10.1128/MCB.26.8.2999-3007.2006 PMC1446934

[b12] Qian M. X. . Acetylation-mediated proteasomal degradation of core histones during DNA repair and spermatogenesis. Cell 153, 1012–1024 (2013). 23706739 10.1016/j.cell.2013.04.032 PMC3983474

[b13] Orgebin-Crist M. C. Studies on the function of the epididymis. Biol Reprod 1, Suppl 1, 155–175 (1969). 5406325 10.1095/biolreprod1.supplement_1.155

[b14] Hecht N. B. Regulation of ‘haploid expressed genes’ in male germ cells. J Reprod Fertil 88, 679–693 (1990). 2182846 10.1530/jrf.0.0880679

[b15] Hermo L., Pelletier R. M., Cyr D. G. & Smith C. E. Surfing the wave, cycle, life history, and genes/proteins expressed by testicular germ cells. Part 4: intercellular bridges, mitochondria, nuclear envelope, apoptosis, ubiquitination, membrane/voltage-gated channels, methylation/acetylation, and transcription factors. Microsc Res Tech 73, 364–408 (2010). 19941288 10.1002/jemt.20785

[b16] Murata S. . Growth retardation in mice lacking the proteasome activator PA28gamma. J Biol Chem 274, 38211–38215 (1999). 10608895 10.1074/jbc.274.53.38211

[b17] Ghyselinck N. B., Jimenez C., Lefrancois A. M. & Dufaure J. P. Molecular cloning of a cDNA for androgen-regulated proteins secreted by the mouse epididymis. J Mol Endocrinol 4, 5–12 (1990). 2322385 10.1677/jme.0.0040005

[b18] Perry A. C., Jones R., Niang L. S., Jackson R. M. & Hall L. Genetic evidence for an androgen-regulated epididymal secretory glutathione peroxidase whose transcript does not contain a selenocysteine codon. Biochem J 285(Pt 3), 863–870 (1992). 1386734 10.1042/bj2850863 PMC1132876

[b19] Lefrancois A. M., Jimenez C. & Dufaure J. P. Developmental expression and androgen regulation of 24 kDa secretory proteins by the murine epididymis. Int J Androl 16, 147–154 (1993). 8514427 10.1111/j.1365-2605.1993.tb01168.x

[b20] Girouard J., Frenette G. & Sullivan R. Comparative proteome and lipid profiles of bovine epididymosomes collected in the intraluminal compartment of the caput and cauda epididymidis. Int J Androl 34, e475–486 (2011). 21875428 10.1111/j.1365-2605.2011.01203.x

[b21] Hall L., Williams K., Perry A. C., Frayne J. & Jury J. A. The majority of human glutathione peroxidase type 5 (GPX5) transcripts are incorrectly spliced: implications for the role of GPX5 in the male reproductive tract. Biochem J 333(Pt 1), 5–9 (1998). 9639555 10.1042/bj3330005 PMC1219548

[b22] de Graaf N. . PA28 and the proteasome immunosubunits play a central and independent role in the production of MHC class I-binding peptides *in vivo*. Eur J Immunol 41, 926–935 (2011). 21360704 10.1002/eji.201041040 PMC3100532

[b23] Otoda T. . Proteasome dysfunction mediates obesity-induced endoplasmic reticulum stress and insulin resistance in the liver. Diabetes 62, 811–824 (2013). 23209186 10.2337/db11-1652 PMC3581221

[b24] Yu G. . Comparative analysis of REG{gamma} expression in mouse and human tissues. J Mol Cell Biol 2, 192–198 (2010). 20494959 10.1093/jmcb/mjq009 PMC2915620

[b25] Thompson W. E., Ramalho-Santos J. & Sutovsky P. Ubiquitination of prohibitin in mammalian sperm mitochondria: possible roles in the regulation of mitochondrial inheritance and sperm quality control. Biol Reprod 69, 254–260 (2003). 12646488 10.1095/biolreprod.102.010975

[b26] Sutovsky P. . A putative, ubiquitin-dependent mechanism for the recognition and elimination of defective spermatozoa in the mammalian epididymis. J Cell Sci 114, 1665–1675 (2001). 11309198 10.1242/jcs.114.9.1665

[b27] Noblanc A. . Glutathione peroxidases at work on epididymal spermatozoa: an example of the dual effect of reactive oxygen species on mammalian male fertilizing ability. J Androl 32, 641–650 (2011). 21441427 10.2164/jandrol.110.012823

[b28] Chabory E. . Epididymis seleno-independent glutathione peroxidase 5 maintains sperm DNA integrity in mice. J Clin Invest 119, 2074–2085 (2009). 19546506 10.1172/JCI38940 PMC2701883

[b29] Pickering A. M. & Davies K. J. Differential roles of proteasome and immunoproteasome regulators Pa28alphabeta, Pa28gamma and Pa200 in the degradation of oxidized proteins. Arch Biochem Biophys 523, 181–190 (2012). 22564544 10.1016/j.abb.2012.04.018 PMC3384713

[b30] Toyoda Y., Yokoyama M. & Hoshi T. Studies on the fertilization of mouse eggs *in vitro*. I. *In vitro* fertilization of eggs by fresh epididymal sperms. Jpn. J. Anim. Reprod. 16, 147–151 (1971).

