# Supplementary material for: Proteasome activators, PA28γ and PA200, play indispensable roles in male fertility
Source: Sci Rep. 2016 Mar 22;6:23171. doi: 10.1038/srep23171 (PMC4802386; doi:10.1038/srep23171)
Supplement: Supplementary Information [file srep23171-s1.pdf]

Supplementary Information for

Proteasome activators, PA28γ and PA200 play indispensable roles in male fertility

Lin Huang[1, 2, *], Kousuke Haratake[1], Hatusmi Miyahara[1], and Tomoki Chiba[1*]

[1]Graduate School of Life and Environmental Sciences, University of Tsukuba, Tsukuba, Ibaraki 305-8577, Japan

[2]Department of Pathophysiology, Dalian Medical University, Dalian, Liaoning 116044, P.R. China

[*]: To whom corresponde should be addressed:
Tomoki Chiba
E-mail: tchiba@biol.tsukuba.ac.jp
Lin Huang
E-mail: lhuang@dmu.edu.cn

Supplementary Table 1 Numbers of offsprings resulting from *Psme3*$^{-/-}$*Psme4*$^{+/-}$ breeding pairs

Genotype	Number
Psme3$^{-/-}$ *Psme4*$^{+/+}$	17
Psme3$^{-/-}$ *Psme4*$^{+/-}$	38
Psme3$^{-/-}$ *Psme4*$^{-/-}$	16

Supplementary Table 2 PCR primers for genotyping

primer	sequence
Psme3	TGGAGACGGCATTGGAGA(Forward)<br>GAACAGAGAGAGCAACTTAACACC (Reverse for wild type allele)<br>AACTCGTCAAGAAGGCGATAGA (Reverse for mutant allele)
Psme4 (for wild type allele)	GGAGACCTTCTGCACTTCCAAGGATCTCAT (Forward)<br>CCTCCCAAGTGTCTAAAGCCGCTTATACTG (Reverse)
Psme4 (for mutant allele)	TCGTGCTTTACGGTATCGCCGCTCCCGATT (Forward)<br>GGATAATATCTCTGCAACAGAAGTTATATG (Reverse)

Supplementary Figures

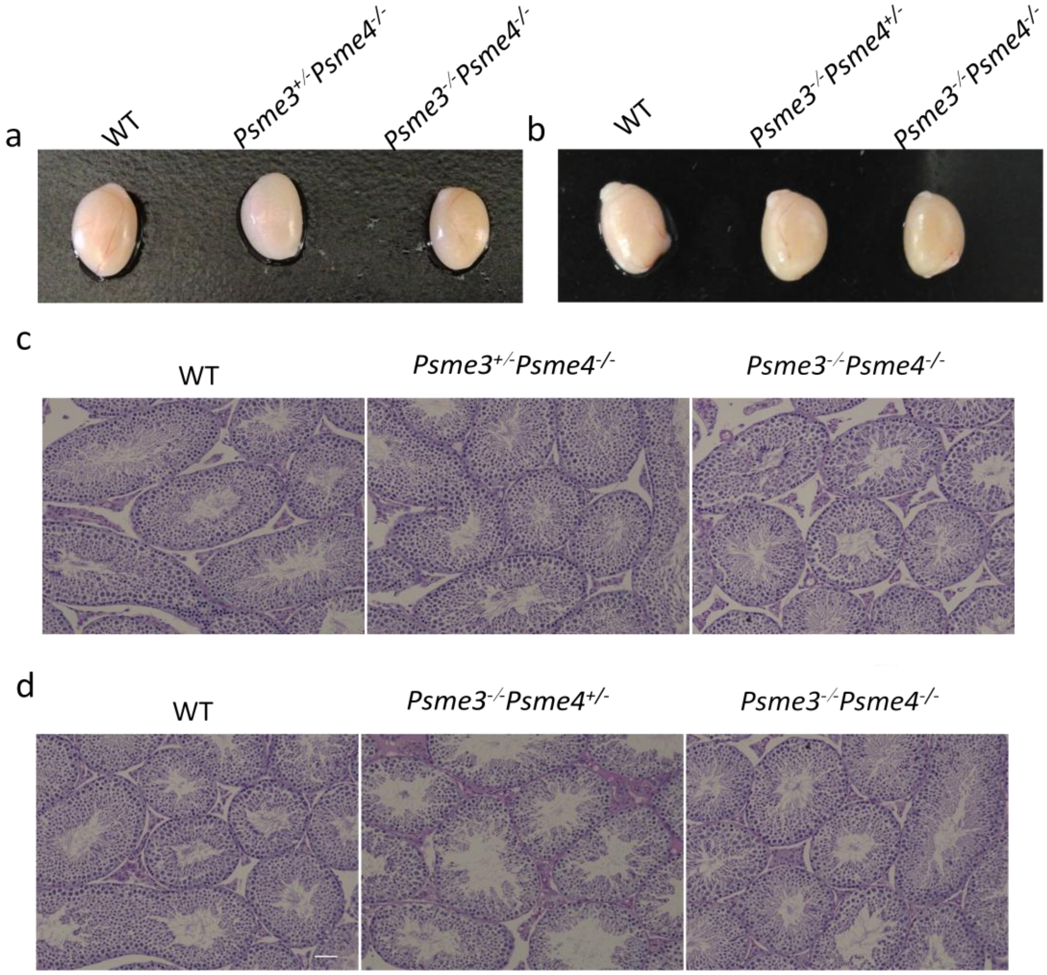

Supplementary Figure S1. Histology of testes.

(a) and (b) Gross view of the testes from wild type (WT), *Psme3*$^{+/-}$*Psme4*$^{-/-}$ and *Psme3*$^{-/-}$*Psme4*$^{-/-}$ mice (a), wild type (WT), *Psme3*$^{-/-}$*Psme4*$^{+/-}$ and *Psme3*$^{-/-}$*Psme4*$^{-/-}$ mice (b). (c) and (d) The PAS-Hematoxylin staining of the testes in (a) and (b). Scale bars, 50 μm.

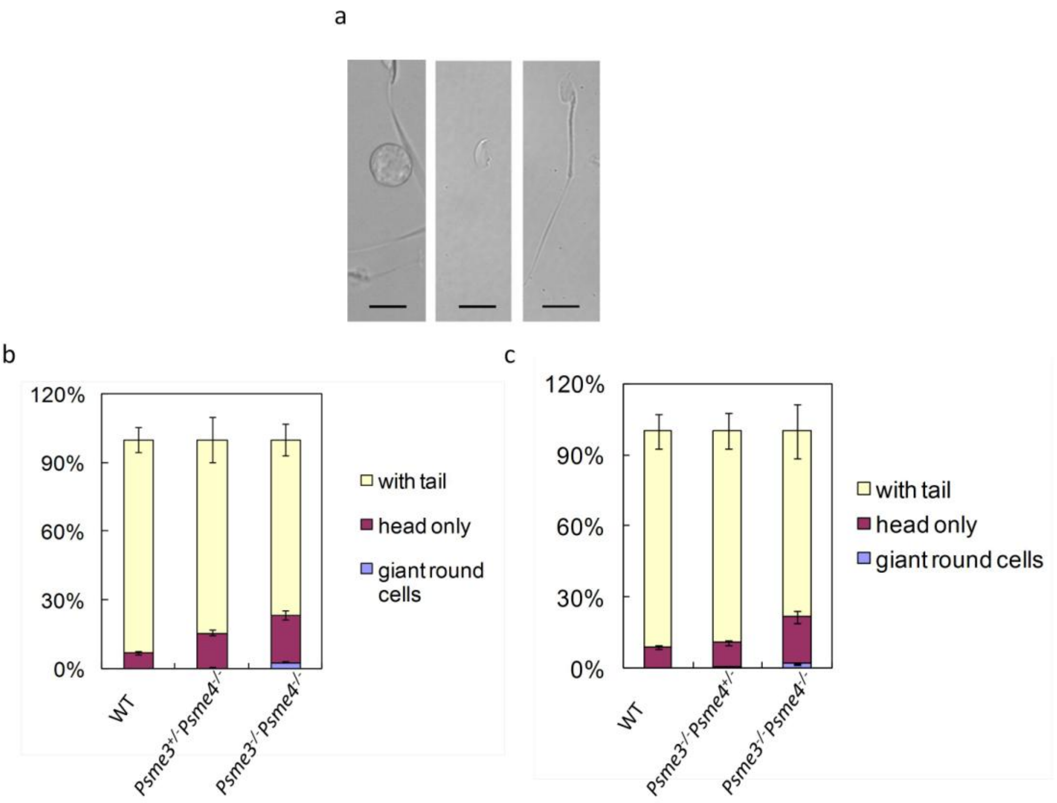

Supplementary Figure S2. Sperm morphology.

(a) Representative images of sperm with tail, head only, and giant round cells. Sperm were collected and observed under microscope. Scale bars, 10 μm. (b and c) The ratios of abnormal sperms for each genotype. 200 sperms were observed per genotype and classified into three groups according to the morphology shown in (a). Sperms were obtained from wild type (WT) (b, c), *Psme3*$^{+/-}$*Psme4*$^{-/-}$ (b), *Psme3*$^{-/-}$*Psme4*$^{+/}$ (c) and *Psme3*$^{-/-}$*Psme4*$^{-/-}$ (b, c) mice. WT is an age-matched male control. The experiments were repeated three times. Bars, BD.

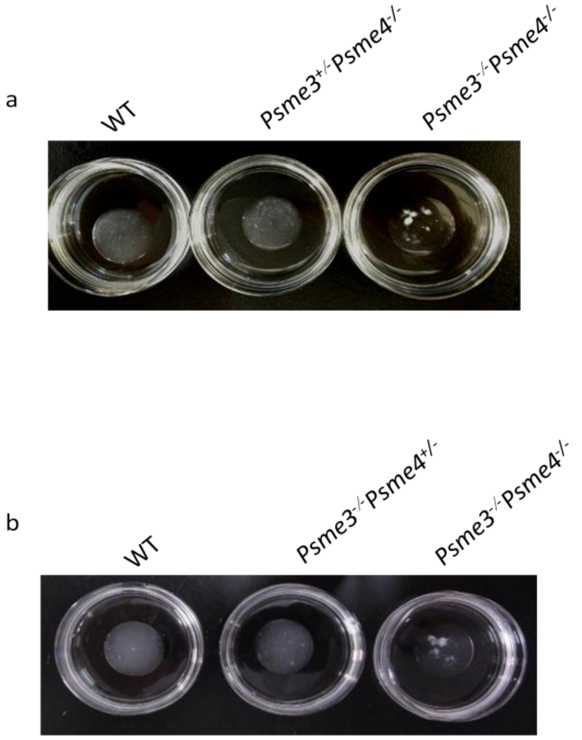

Supplementary Figure S3. Sperms from cauda epididymis. Sperms isolated from the cauda epididymis of wild type (WT) (a, b), *Psme3*$^{+/-}$*Psme4*$^{-/-}$ (a), *Psme3*$^{-/-}$*Psme4*$^{+/-}$ (b), and *Psme3*$^{-/-}$*Psme4*$^{-/-}$ mice (a, b) were incubated in TYH drops for 1 h.

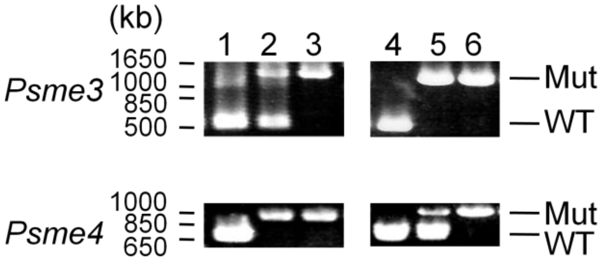

Supplementary Figure S4. Genotyping of *Psme3/Psme4* dKO mice.

The wild-type (WT) and mutant (Mut) alleles of *Psme3* (upper panels) and *Psme4* (lower panels) were PCR amplified. Typical results of wild-type (lanes 1, 4), *Psme3*$^{+/-}$*Psme4*$^{-/-}$ (lane 2), *Psme3*$^{-/-}$*Psme4*$^{-/-}$ (lanes 3, 6) and *Psme3*$^{-/-}$*Psme4*$^{+/-}$ mice (lane 5) are shown.

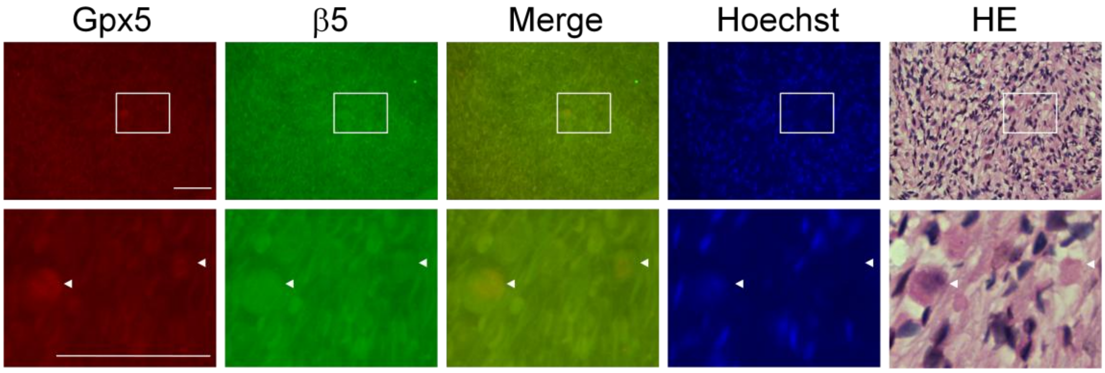

Supplementary Figure S5. High expression of Gpx5 in the cauda epididymis of *Psme3/Psme4* dKO mice.

Immunohistological and HE stainings in the same section of the cauda epididymis. Scale bars, 25 μm. Arrow heads, strong Gpx5-positive signals. Lower panels show higher-magnification images of the insets in upper panels. Red, Gpx5; Green, 20S proteasome β5 subunit; Merge, the merged images of red and green; Blue, Hoechst 33342.

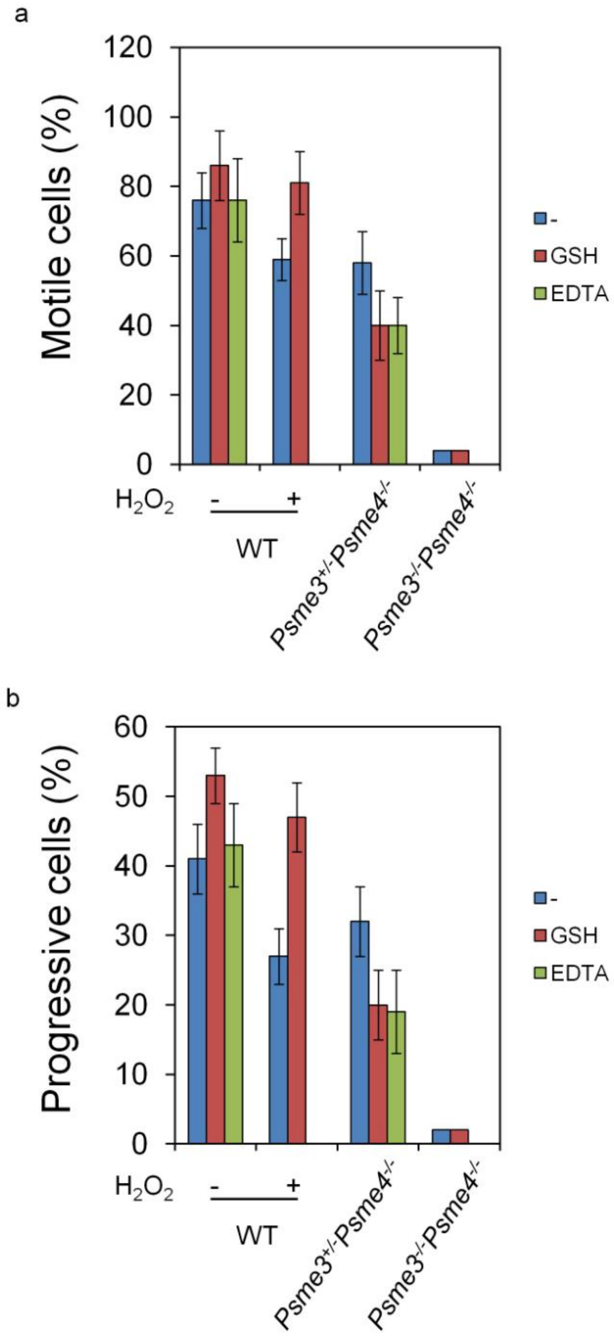

Supplementary Figure S6. The effect of antioxidant on sperm motility.
Sperms isolated from the cauda epididymis of wild type (WT), *Psme3*$^{+/-}$*Psme4*$^{-/-}$, and *Psme3*$^{-/-}$*Psme4*$^{-/-}$ mice were incubated in TYH drops with GSH (10mM) or EDTA (100μM) for 1.5 h. The WT sperms were also treated with H_2O_2 (100μM) in the presence or absence of GSH (10mM). The percentages of motile cells (a) and progressive cells (b) in each genotype were analyzed by CASA. Bars, SD.
